# Stakeholders' Viewpoints on Working to Advance Health Equity

**DOI:** 10.1089/heq.2023.29040.rtd

**Published:** 2024-01-08

**Authors:** Laurie Zephyrin, Morenike Ayo-Vaughan, Andrew Bossick, Amanda Noroña-Zhou, Eve Higginbotham, Molly Richardson, Hector Rodriguez, Allison Bryant

**Affiliations:** ^1^ Senior Vice President, Advancing Health Equity, The Commonwealth Fund, New York, New York, USA.; ^2^ Program Officer, Advancing Health Equity, The Commonwealth Fund, New York, New York, USA.; ^3^ Assistant Scientist, Henry Ford Health, Detroit, Michigan, USA.; ^4^ Assistant Director of Developmental Medicine, University of California, San Francisco, California, USA.; ^5^ Vice Dean for Inclusion, Diversity, and Equity, University of Pennsylvania Perelman School of Medicine, Philadelphia, Pennsylvania, USA.; ^6^ Visiting Assistant Professor, University of Alabama at Birmingham School of Public Health, Birmingham, Alabama, USA.; ^7^ Kaiser Permanente Endowed Professor of Health Policy and Management, University of California, Berkeley, School of Public Health, Berkeley, California, USA.; ^8^ Maternal-Fetal Medicine Specialist, Associate Chief Health Equity Officer, Massachusetts General Hospital, Boston, Massachusetts, USA.

## Introduction

I would like to welcome everyone to: *A Glimpse at How Stakeholders Are Working Towards Achieving Health Equity Expert Panel Discussion*. My name is Morenike Ayo-Vaughan and I am the Program Officer of the Advancing Health Equity program at the Commonwealth Fund.

While the Fund has historically supported efforts focused on eliminating health inequities, the Advancing Health Equity Program was formally established in 2021 with the goal of identifying practical solutions to eliminate racial and health inequities within health systems and policy. As a part of this effort, we are excited to support the development of this important special focused collection of articles published in the *Health Equity* journal, and we thank you for your manuscript submissions.

With increased focus on health equity research, we believe it is important to capture results and solutions so that they can be shared with other healthcare stakeholders seeking to promote health equity within their organizations. I would like to thank each of you for joining us today to share additional insights regarding your work. I am joined by Dr. Laurie Zephyrin, Senior Vice President-AHE who will be moderating our first panel.

***Dr. Laurie Zephyrin:* Dr. Bossick, I wanted to talk a little bit about your paper. It touched on the ability of state policies to enable or restrict reproductive autonomy.^1^ Can you please explain what reproductive autonomy entails, and how is this reflected in state policies?**

**Dr. Andew Bossick:** Reproductive autonomy is a person's ability or power to make and act on decisions about contraceptive use, pregnancy, and childbearing. Essentially, having full personal decision-making control on whether or not to have children. And, if you have children, the timing of when you have children, as well as the spacing of those children. It is important to note that within the United States, there is a strong history of reproductive oppression, especially among people of color (POC)—much of which still exists today—which has devastating and long-lasting effects on reproductive autonomy.

***Dr. Zephyrin:* How does this impact the disparities we see in preterm and low-birth weights, which is what your paper was assessing?**

**Dr. Bossick:** We found that more restrictive policies were related to worse outcomes for preterm birth and low-birth weight. We see that broadly for other restrictive policies in other studies.^2–8^ Policies that support reproductive autonomy, however, may narrow the disparities we see in these outcomes, and in my mind, for one major reason: The reproductive autonomy of POC, especially, has consistently been shown to be limited more than other populations–primarily, white populations. From forced sterilization, to pushing the prescription of long-acting reversible contraception, to the vast literature that says that POC voices are less likely to be heard, respected, and listened to in a medical setting. If we create legislation centered on the experiences of those most affected, POC especially, I expect birthing outcomes in those populations to improve.

***Dr. Zephyrin:* Dr. Noroña-Zhou, your article examined ethnic and racial disparities in preterm-birth and low-birth weight before and after the implementation of a trauma-informed model of care.^9^ What does the trauma-informed model of care entail and how might this model be scaled to other health systems?**

**Dr. Amanda Noroña-Zhou:** I would like to start by contextualizing the study a little bit for the purposes of our discussion today. We implemented a trauma-informed approach to care within an adolescent maternity program. This is a unique population that might particularly benefit from a trauma-informed approach, as this group is predominantly young pregnant POC, a group that experiences high levels of stigma, racism, and discrimination across many settings, particularly in health care.

The idea here is that improving a health care system—in this case, a clinic for pregnant adolescents—represents a significant effort that could benefit two generations. With that context in place, I want to mention that co-authors on the current paper published another paper in 2018^10^ that describes in detail the specific changes that were made in the adolescent maternity program to integrate a trauma-informed approach. That paper is by Drs. Bethany Ashby, Amy Ehmer, and Stephen Scott and I highly recommend it as a resource for anyone interested in the nuts and bolts of the implementation. In our specific clinic, the departments of Psychiatry and OB/GYN received an endowment from a private foundation which came with a directive to collaborate to provide high quality behavioral health services to perinatal women. Given the Substance Abuse and Mental Health Services Administration's (SAMHSA) efforts in behavioral health integration, the Adolescent Maternity Program's leadership use their Six Key Principles of a Trauma-Informed Approach as the template for how to optimize the support offered in the Adolescent Maternity Program. SAMHSA defines a trauma-informed approach as a program, organization, or system that is trauma-informed, realizes the widespread impact of trauma, and understands potential paths for recovery. It recognizes the signs and symptoms of trauma in clients, faculty, families, staff and others involved within the system, and responds by fully integrating knowledge about trauma into the system's policies, procedures, and practices. And this is the critical piece: it seeks to actively resist re-traumatization.

The six key principles that SAMHSA advocates in their approach are first, safety. Second, trustworthiness and transparency. Third, peer support. Fourth, collaboration and mutuality. Fifth, empowerment, voice, and choice. And sixth, historical, cultural, and gender issues.

A couple of quick examples of how those key principles are translated into real-world clinical changes. The key principle of safety—how the Adolescent Maternity Program embodied changes related to psychological safety—were that we made every effort to ensure that medical care was provided by the same provider throughout pregnancy. So instead of the general standard of care of pregnant folks come in and see whichever provider is available on the schedule that day, every effort is made to have continuity in medical care as well as behavioral health support.

In addition, people have circumscribed roles to avoid duplication of services, particularly related to medical history and trauma history so that patients were not required to retell their life experiences. One of the changes made in the Adolescent Maternity Program was to see every patient who came into clinic, no matter how late they were to their appointment, no matter how many visits they had missed in the past. Patients were not terminated from services based on their rates of no-show appointments. We provided free transportation for patients if they could not afford transportation on their own. We encouraged folks to bring their children into the visits with them if childcare was not available. We used holistic approaches to embody a trauma-informed approach in adolescent maternity care.

The second piece of your question of how to scale remains a really important and largely under-attended to next stage of this work. Given my description of this trauma-informed overhaul, I am sure you can imagine the amount of money, time, advocacy, and dedication across the whole team that this effort took. I think, critically, it also involved taking a big step back from care as usual, and taking a moment to reflect and pinpoint those standard medical practices that could be retraumatizing to our patients.

These clinic-level changes were not easy, fast, or inexpensive, but they really paid off in terms of health outcomes, which is the outcome that we should all care about the most. In terms of how to scale, this involves advocacy on the part of medical systems leveraging the power that they have with the federal and state government, and with insurance companies, to fund clinic overhauls and adjustments like this one. Also, advocating for better reimbursement of mental health services across the board.

***Dr. Zephyrin:* It is fantastic to hear that you were able to apply and implement this trauma-informed approach in this context. We know that trauma-informed care can have huge benefits. It is really important to see this linkage around maternal health and health equity, particularly in this adolescent population. Thank you, Dr. Noroña-Zhou.**

**Dr. Richardson, your paper really sought perspectives from obstetric health care providers, from health administrators, from local organizations, all of whom provide pregnancy, delivery, and postpartum care services in Alabama, to understand why racial disparities persist.^11^ I commend you for truly highlighting the experiences of community members and the importance of collaborating with community members. From your experience in doing this work, how best can policymakers meaningfully engage community members when developing policies?**

**Dr. Molly Richardson:** From our experience and understanding of the evidence, community engagement is essential to identify, understand, and to begin to address these challenges. Developing and sustaining relationships is key, although this may be time-consuming and require considerable investment from all parties. Working with existing partners is key to identifying the correct stakeholders, people with shared interest and investment, to engage with.

It is also important to acknowledge the spectrum of diversity of interested parties from key informants to long-term partners and decision-makers, and the varied investments that they may be able to contribute over time. This goes a long way in working together to understand and agree to priorities, expectations, communication styles, and timelines. We strive to listen first and implement mixed methods approaches in various contexts. This is often where qualitative work can complement and elevate quantitative surveillance like surveys. For example, public meetings or workshops in spaces where community members are active or paired with existing events can be a great opportunity for listening sessions or other formal prioritization techniques like focus groups. Employing best practices for health literacy in all communication including social media and more traditional avenues of communication like signage, flyers, and other materials. We strive to work collaboratively with trusted resources of information like lay community health advisors and other established informal community leaders.

Engaging those responsible for implementing the strategies, and those who will directly be involved in participating in those strategies, are key for buy-in, feasibility, and relevance. In particular, community advisory boards (CABs) can be helpful in establishing relationships. CABs can develop agreements on shared and transparent decision-making processes. Compensating community experts with flexibility for the time, travel, and knowledge of their lived experience is essential. This can help to avoid repeating an issue of historically undervaluing these resources.

Finally, the strategies I've described are informed by existing resources, like Community Engagement: A Practitioner's Guide,^12^ The Community Guide,^13^ Principles of Community Engagement,^14^ and many other evidence-based sources that can guide our work. Most importantly, tailoring these to the unique context and partners is essential to inclusion of all those involved and engaged in this work. Service providers have suggested strategies including policies that should be community-focused, evidence-based, and culturally sensitive. It is with effective policymaker engagement with communities that we can move in the direction of health equity.

***Dr. Zephyrin:* Thank you for sharing those important insights. I have a question for the three of you. Part of what we wanted to do in terms of highlighting this work is really to highlight some of the solutions around policy in system and community level. What are opportunities that you think, based on your work and what you have observed, to develop or modify policies to advance maternal health equity?**

**Dr. Bossick:** I think something that comes top of mind would be a policy to legislate expanded benefit coverage for mental health care, that includes mental and behavioral health coverage, to at least last at least 1 year post-delivery, given that mental health conditions are major drivers of maternal morbidity and mortality.

**Dr. Noroña-Zhou:** I could not agree more with Dr. Bossick. But another idea is advocating for caregiver paid leave policies. I think it is a huge problem when folks who have just had a baby are required to go straight back to work to make ends meet. I think that advocating for leave and time to recover, with some financial cushion, is critical.

**Dr. Richardson:** I'm grateful and in agreement with the other panelists. The people living and working in this space shared specific actionable strategies that could be applied at systems and policy levels. States that have not already should elect to expand Medicaid. Alabama is one of 10 states that has not yet elected to do this. Focus on adequate health insurance. Remove chemical endangerment laws that criminalize pregnant individuals. Support substance use and mental health support programs. Remove laws that restrict choice, whether that's choice of provider, institution, or maternity services. Include diverse, interested parties in policy making at all levels. This should include not only racial and ethnic diversity, but diversity of experience, including varied career disciplines and change organizations. I appreciate some of these are particularly relevant to the Deep South, but there is greater reach as well.

***Dr. Zephyrin:* You also highlight the importance of the policy piece as well. Dr. Bossick, I was struck by your article in terms of really looking at reproductive policies and the impact on policies that can promote equity, or policies that can widen disparities. I was very interested that you were able to develop this index of reproductive autonomy. Do you think there are wider uses of this work in terms of creating measures that can help determine whether policies are equitable or not?**

**Dr. Bossick:** I think so, and I think that it is required. I think all state policy that is being proposed should go through some sort of equity evaluation to determine the effect that it will have on certain communities, as well as how much that it is going to cost and how it is going to affect the delivery of health care services.

***Dr. Zephyrin:* Absolutely. Before I close this segment, I am just so intrigued by all of your work and its policy and systems implications. I would love a final, closing thought from each of you in terms of the research that you have done and the next steps you see it taking.**

**Dr. Richardson:** We are so proud to continue this work that we are doing, and to continue the relationships with partners that we have built. We are currently working as a part of the American Heart Association's Health Equity Research Network (HERN) on Disparities in Maternal-Infant Health Outcomes called the P3 EQUATE Network. Specifically, P3 Providing an Optimized and emPowered Pregnancy for You, or POPPY^15^ pilots some of the strategies that were suggested by our clinical and complementary providers to engage pregnant individuals who are Black and African American with community health workers and digital health interventions during the pregnancy and postpartum period. We look forward to sharing that as we learn and grow.

**Dr. Bossick:** I am proud and humbled that I am able to do this research and work with communities, especially the index that we created was working with communities to develop it, was important. I think that needs to be more integrated into additional research strategies. Future work that we have is around persons with disabilities and how they navigate maternity care. I think that should be a priority; I know that the CDC has made it a health disparity officially. But it needs to have some research dollars that back it so that we can really dig into what people are experiencing.

**Dr. Noroña-Zhou:** In California at least, where I am based, we are noticing that this moment in time is a tide shift in terms of collective public interest and policymaker interest in maternal and child health. I think it is very exciting to be part of this panel and being a piece of that puzzle. One of the next directions of our work is looking into additional clinical adjustments that can be made to improve health outcomes and specifically, move toward health equity. One project that is in the works right now is looking at racially concordant primary care for Black families in Oakland (California). There are huge known benefits of racially concordant care in the prenatal and perinatal period, and I think bringing that into pediatric primary care is the next frontier. We are excited to see where that goes.

***Dr. Zephyrin:* Thank you; that is fantastic. Again, as you can hear from this panel, as we think about driving change that advances maternal health equity more broadly, we must really understand and think about systems and policy initiatives that are so critical. And really integrating community voices into the work, from its inception, whether we are talking about developing policies and characterizing equity impact of policies, whether we are doing change at the systems level from trauma-informed care to other models of care, or whether we are really understanding how communities can lead care with policy and systems as well. So, thank you all for your work; I am so excited to see it continue. And for all of you that are reading, please go to the *Health Equity* journal to read their research articles. They are profound, and I believe they have a great deal of opportunity to continue to drive policy and systems change.**

**Ms. Morenike Ayo-Vaughan:** Our motivation for this panel is to learn in the real world. What are health systems doing to operationalize health equity? We know that a lot of systems have made promises and pledges, and we are very excited to read these articles that are talking about how these are being applied in a practical sense. Thank you to each of you for joining us today.

My first question is for Dr. Eve Higginbotham. From your article, we learned that your health system deployed a Whole-Scale Strategic Planning process as part of its strategy to foster cultural transformation.^16^ My first question is regarding which aspects of this process would you say were most meaningful in advancing a culture of addressing racism within the institution?

**Dr. Eve Higginbotham:** This is a great question and one that I would like to first answer by acknowledging the creators of this process. Dannemiller and associates described this process^17^ years ago and I have used this process organizationally in my current and previous positions. We chose this process, as described in our paper. In this instance, it was particularly helpful because of the sense of urgency to respond to the community and initiate change that was generated following the murder of George Floyd in the summer of 2020. What was unique in our application of whole scale strategic planning was the use of a virtual platform, which was required at the time given the limitations on group meetings and masking associated with pandemic.

One of the key elements of this process is to everyone coming together around a common purpose and to listen to as many voices in the processes. And certainly, the common purpose was a need for a change and address structural racism within the organization. We must change because the culture is impacting individuals within the community of our organization differently, so the common purpose was already set. The other feature of this process is that it emphasizes developing the microcosm of the whole, bringing together key representatives from just about every piece of the organization. That was critically important because everyone is impacted differently. Bringing so many stakeholders together in this process also created that opportunity to filter up as many of the best recommendations that we could glean, because form the outset, there were more than 150 we had several recommendations offered by individuals and groups across the enterprise. Actually, in the hundreds, as you can imagine. I think every health system was being bombarded by calls for change from key stakeholders amid the pandemic. The whole scale strategic planning process provided a path forward for us to synthesize the broad input we were receiving, but it gave us, at least, a process by which we could create themes, as well as develop priorities, and succinctly articulate the input we received to leadership. Speaking of leadership, the other key feature to highlight is the is that we had alignment of leadership–and I cannot emphasize that enough—because even though we are an integrated health system where the dean is also over the health system as well, you still have very different cultures within the School of Medicine versus the health system.

There was clear alignment between the CEO of the health system, as well as the dean. That alignment was critically important. I think that is the other feature of the whole scale process, is that you had buy in from other levels of leadership, besides the most senior and the most junior.

We also developed a very discreet governance model so that there were layers of individuals who would sign off on these recommendations. Lastly, clear and interactive communication is required to ensure that those who provided the initial input know that their voices are being heard. I think that is the other feature of the whole scale process, is that you had buy in from other levels of leadership, besides the most senior and the most junior.

**Ms. Ayo-Vaughan:** Dr. Bryant, your health system launched a systemwide initiative to become an anti-racist health care organization, United Against Racism. Can you please walk us through the three pillars that comprise this initiative?

**Dr. Allison Bryant:** United Against Racism, or UAR, as we affectionately call it, was launched in October of 2020, systematizing work that was already being done in each of our hospitals. Mass General Brigham is a large healthcare organization in Massachusetts and New Hampshire that includes 12 hospitals that are academic medical centers, community and specialty hospitals and a number of outpatient practices.

So much of this equity work had been ongoing in these organizations, but really, this was our attempt to move toward anti-racism by creating some core values for the larger health system. United Against Racism is a multi-million-dollar platform that essentially thinks about three pillars of work. The first is thinking about our leadership, our employees, and our workforce culture, and that lives under our human resources and our DE&I teams. But clearly, all these layers intersect; those workstreams include things like developing an enterprise-wide anti-racism education. We have 82,000 employees across our organization. You cannot work here unless you have done our Stepping Stones Training, as well as an additional anti-racism training that was developed. Our workforce may speak many languages; English may not be the primary language. And particularly during COVID, how were we getting those messages to our employees in the languages that they preferred? We are also thinking about our leadership and making sure we are retaining diverse leaders.

The work that we do under the Office of the Chief Medical Officer really is in thinking about our patient and community care. How do we ensure that we have equitable patient care experiences in all of the places that we serve our patients and how do we think about achieving equitable outcomes for the communities that we serve? Even if individuals are not Mass General Brigham patients, we do feel very much a responsibility to ensure equity for our communities. And so that work includes foundational things, like ensuring appropriate data accuracy, thinking about language accuracy. How do we make sure that the written information that we are giving to our patients, the ways that we are communicating with them verbally, are in the languages they prefer? And then thinking about digital care and the fact that we have now done so much in terms of virtual care, but still understanding that digital literacy is not evenly distributed among our population. So, standing up things like digital access coordinators to provide at-the-elbow support for those who need it most.

Also, consideration that many of our clinical policies have racism baked into them. So how do we look for those policies systematically and review them with that equity lens? And then change those responsible for excess mortality for Black and Brown individuals, and how do we add extra resources to individuals with uncontrolled hypertension, for example, or who are at risk for cesarean delivery, to ensure the best outcomes?

Lastly, in our community-facing pillar, how do we use our voices and organization to advocate for change at the local, federal, legislative levels? How do we deliver the care that we provide in our brick-and-mortar sites out in our communities, by things like community care vans and others? So that has been the work of the past 3 years of United Against Racism, which hopefully, we will build on going forward.

**Ms. Ayo-Vaughan:** Thank you for sharing. It is great to see common themes between the work you are doing in your system, as well as Dr. Bryant and the work Dr. Higginbotham is also leading. Switching gears a bit, I am going to turn it over to Dr. Rodriguez, whose work is focused on speaking to providers who are participating in a pay-for-equity program in the commercial payer space.^18^ Through your research and speaking to providers that are participating in this program, what would you say are the biggest facilitators to providers incorporating these pay-for-equity requirements?

**Dr. Hector Rodriguez:** I would like to highlight five facilitators that we found to be very important. The first one is a confidential reporting-only period to acclimate provider groups to the equity performance standards and to really be able to have a baseline understanding of how enduring the disparities are for the various clinical performance measures.

In this case, it is the Healthcare Effectiveness Data and Information Set (HEDIS) performance measurement set. This enables the group to really understand the gaps they need to close before being incentivized. They had 3 years of Blue Cross Blue Shield of Massachusetts feeding back information from 3 years of performance data to give the groups a very strong understanding of racial inequities in their system, before incentivizing them to improve it.

The second piece that I think was a true facilitator for the health plan was to build on its existing value-based payment program called the Alternative Quality Contract (AQC) which has already incentivized quality improvement with a budget constraint. The evidence indicates that the AQC improved quality of care in these HEDIS measures, while also controlling the rate of cost growth over 8 years. Providers bought into a lot of these components of the AQC which has enabled them to understand the equity incentives in the context of that existing program, so modifications to the program in terms of the incentive formula were made. Otherwise, the program is very similar to the AQC, so it really facilitated buy-in.

The third thing is that Blue Cross Blue Shield was wise to establish a collaborative learning system called the Equity Action Community. As you know, there are emerging evidence-based practices to reduce racial inequities in quality of care, but the field is still nascent, and more evidence needs to be generated. As a result, innovation is going to be required to address these racial inequities and quality of care. We need to expect some failure. Part of the learning process is to identify and share the practices and the policy and organizational changes that were tried. Having the Institute for Healthcare Improvement as the expert convener for this work has been key to provider engagement. Blue Cross Blue Shield of Massachusetts is currently building their own capacity to convene and support identification and dissemination of effective equity-focused interventions. This is the capability that I think a lot of health plans who are going into this space will need to develop.

The fourth enabling condition or facilitator was upfront investment in infrastructure to improve equity. A number of groups have started to embark on this initiative but have very little experience addressing racial inequities. And while MassHealth, the Medicaid payer in Massachusetts, has a very robust equity initiative for Medicaid beneficiaries, Blue Cross Blue Shield of Massachusetts was the only payer making upfront infrastructure investments, so what we see is this is really facilitating engagement. But actions from other payers, and commercial payers, are likely to be needed currently.

Blue Cross Blue Shield is using a payer agnostic approach to these investments and allowing groups to not just focus on their commercially insured patients from the health plan, but also other commercially insured patients and their Medicaid patients. I believe the equity infrastructure investments from all payers will likely be needed moving forward.

The fifth and final facilitator is the alignment of equity measurement and reporting requirements across payers. MassHealth and Blue Cross Blue Shield of Massachusetts are beginning to have alignment in their measurements which has facilitated groups to take action. When there are many different requirements, it is very hard to move equity initiatives forward, so having standardization across payers has facilitated progress.

**Ms. Ayo-Vaughan:** Thank you, Dr. Rodriguez. It is good to see the synergies between what systems are doing and what payors are doing. One thing we have learned in our work here at The Fund is the importance of data, race, and ethnicity data collection, understanding payer mix. I would like to pivot a bit to learn more about the types of data you are using to inform the different work that you do.

Dr. Higginbotham, we read in your article that there was an implementation of a balanced scorecard. Health systems are trying to figure out what type of scorecard they should be creating regarding health equity. Would you please share more about the tool, how it was used, and what changes were made based on the results of the scorecard?

**Dr. Higginbotham:** Like the whole scale strategic planning process I mentioned earlier, the balanced score card^19^ is another tool which I have found helpful throughout my career. The balanced score card provides a means to track leading indicators such as infrastructure and processes and lagging resources, such as financial stewardship and stakeholder satisfaction. One of our key metrics is in the assessment of the culture of the institution, as a means to assess stakeholder satisfaction. We have used something called the Diversity Engagement Survey. That survey has allowed us to provide an opportunity for respondents to self-report how they want to be identified. We also track health equity goals such as maternal morbidity, as a means of assessing how well we are serving the community. I believe a key element is asking people to self-report.

We have other demographic identifiers as well, such as facets as well. We look at religion, geography, and sexual orientation. There we look at geography in terms of where they are from, et cetera., so I think it is a more granular demographic characterization that we are seeking, not only for our patients, but also our faculty, staff, and students, as they complete their responses for the Diversity Engagement Survey there. We are also looking at sexual orientation as well. On the systems' side, it is similar to that for patient care, as it is with other systems.

This more specific characterization provides the opportunity to develop initiatives and action items that are focused on specific needs. At Penn Medicine, we have now offered individuals the opportunity to be even more specific in terms of their demographics, so there are scores of social identities that can be registered. It is a matter of giving people the opportunity to self-report in the way they would like to be self-identified and providing them the necessary specific actions to address their needs and concerns.

The balanced scorecard has been around for a while, but I have always liked it as a way to follow and monitor strategic planning efforts over time because it gives us an opportunity to balance the leading indicators of organizational infrastructure and processes with lagging indicators of financial stewardship, as well as stakeholder satisfaction. So, in our case, going back to the Higginbotham article as evidence of the need for more of the leading indicators compared to the lagging indicators, when we analyzed our initial action items which we call *Just Do Its*, which were required to be accomplished. Within the first 12 months we found that 31% of the *Just Do Its* were associated with such a need for organizational infrastructure and 42% were categories as well as key processes. Thus, 73% of the initial action items were important to implement so that the results were seeking, represented by lagging indicators will be realized, that more than 70% of our first action items.

I would like to highlight examples of *Just Do Its* because of the importance of identifying immediate actions before developing the strategic framework. As mentioned, *Just Do Its* were items that we knew had to be done, like taking down a picture of an enslaved individual that our medical students had been complaining about for years. Just take it down because it is causing people to be uncomfortable. An example of a *Just Do It* that we would call a process, if you will, for determining what should be hung on our walls. Ultimately there was a committee formed to determine what should be hung on walls going forward.

Another initiative we did immediately was to require unconscious bias training. Over 97% of our 45,000 employees underwent unconscious bias training. We tracked it. We quantified it. And now, new employees must have that as a basic understanding of what is important to work in our space.

Those are just a few examples. The attention to infrastructure and processes, guided by the e balanced scorecard ensures that we are putting the attention where it's we needed to put into organizational infrastructure, as well as key processes to ensure that we have sustainability of our efforts.

**Ms. Ayo-Vaughan:** I think a common theme that we have also found across health systems is the incorporation of better training. And of course, the *Just Do It* attitude. There are short-term wins, always, and short-term solutions that can be implemented.

Dr. Bryant, in line with the work that Dr. Higginbotham's team has been doing, we saw in your article that your health system developed a virtual “Upstander” training, where staff can report instances of racism.^20^ How are these instances being addressed when they are reported? Is there a systemwide analysis happening?

**Dr. Bryant:** Even though our work is somewhat siloed into pillars, we know that this work crosses between them. This work sits in our plan to develop enterprise-wide incident reporting and response, so that lives between human resources and us in the Office of the CMO. It is because we recognize that our health system is just a microcosm of what is happening everywhere.

Instances of racism and discrimination will occur within the many worlds of our health system whether it is from one staff member to another staff member, staff to patient, patient to staff, etc. We wanted to build robust systems to collect and track, and to truly invite people to let us know what was happening so that we could adjudicate and reconcile them. I will say we have a long way to go. This work is very nascent; there is so much we are learning.

But one of the things that we did was to be able to respond to a patient who acts in a way that is discriminatory or racist toward a staff member. We now have a Patient Code of Conduct that guides us, where we can turn to the patient and say, “That is not how we behave in this environment. If you continue to repeatedly violate that, we may terminate our relationship with you. You may not be able to get care within our Mass General Brigham sites” (Supplementary Data). This happens within our safety reporting systems, so many people are familiar with safety reporting in their hospital systems. In several of our hospitals, we now have an icon, the Safety Reporting Platform, that says, “Was this an episode of discrimination or racism?”

If there was a medication safety event, within that context, was there any discrimination or racism at play? Someone can check that off. The idea is that those cases, where racism is flagged, that we should be having a special equity review of those cases and instances. We have the same thing in place if it is staff to staff and it happens within our concerned management system, for example. Then Upstander is a way to make sure that those individuals who are most likely to receive those reports are not racism deniers—that the first thing that does not come out of their mouth is, “That didn't really happen,” and to help them to understand ways that they might help to reconcile some of these events. So far, we have trained about 1300–1400 of our staff members—mostly in managerial roles—but I think that what we are understanding is that staff are just as likely to turn to a peer and let them know that something has happened, and so we really need to broaden out our efforts for Upstander training.

In terms of your question about quantifying, we have not yet built in many robust systems of understanding how this work is being taken up. We do have ways to track the number of safety reports. In those organizations where we have piloted the safety reporting icon, we have seen a dramatic increase. As we have built it, people have come to let us know that these cases are happening. We are hopeful that over time, we will have an increase but then hopefully, a plateauing and even a decrease as we help to adjudicate and build this culture of equity across our organization.

**Ms. Ayo-Vaughan:** Again, I think another theme is the importance of culture change across systems. So, Dr. Rodriguez, through your work in conversations with providers in the outpatient settings, could you share a bit more about what providers report to be the biggest cultural changes they have had to make while participating in these pay-for-equity initiatives?

**Dr. Rodriguez:** I have four areas to discuss around cultural challenges. The first is how to internally manage and support equity work within the health system. We observed a wide range of capabilities with respect to provider groups; how they are situating and supporting and resourcing equity work, from large organizations who have multiple executives and performance improvement professionals embedded in the equity work, to ones where it is 10%–15% of a doctor's job.

I believe that we need to build the evidence and characterize how best to support equity work. I think organizations are struggling with how to do this best and are looking to one another and examples from the field. I think it is a major cultural change in organizations in how to elevate it and how to support it well.

The other one that has been talked about a lot here is the routine collection of race, ethnicity, and language data. This is a key infrastructure to engaging in this work, both to monitor and measure equity, but also to implement targeted and tailored interventions. In the absence of these data, groups have used demographic data to inform targeting, but I have found that this tends to be a barrier to action in terms of not having the perfect data. Other groups have relied on the claims data from the health plan to help them understand their disparities. I think that has been a cultural challenge: the need and the importance to routinely collect race, ethnicity, and language data. We have been taught, as good public health professionals, to think about systems improvement, and that by improving systems, we are going to lift all boats. But lifting all boats means that you are not reducing the inequities, so targeted and tailored interventions are often needed. However, I think groups are banking on system improvement to get them to reduce racial equity. The jury is still out in this initiative, whether that approach is going to truly reduce the inequity, or just keep it the same and lift all boats.

I have not seen as much tailored and targeted interventions to racial and ethnic groups that I would expect. Underscoring that systems improvement is the primary way that the groups are undergoing this change, so that is a challenge.

The other challenge that I have seen in diverse contexts, even though we have a lot of evidence about racial inequity and quality of care, is that there is a tendency for groups to think about other vulnerabilities, including sexual orientation and gender identity. I think that it is important to recognize intersectionality, but not to dilute the focus on advancing racial equity because that is where the starkest disparities exist. I think the discipline to stay focused on race has been a challenge, given the key stakeholders' influences on prioritization.

**Ms. Ayo-Vaughan:** Thank you for that insight. This has been a wonderful conversation. When we think about changes health systems can make, from our experience, we know that both providers and larger health systems are struggling with operationalizing health equity. It is still a conversation they are trying to get on board with, and what we are so appreciative of in all of your submissions is the fact that they are putting this in action. Dr. Rodriguez mentioned tailored interventions. Dr. Noroña-Zhou talked about trauma-informed care delivery. Specifying those type of examples are what we are trying to showcase through this *Health Equity* special issue. Our goal is that through this roundtable, through your discussion, and through this *Health Equity* special issue, that health systems, researchers, and payors out there that are struggling to get on board with health equity can look at each of these examples and take from your recommendations and launch forward. We thank you so much for joining us today and for your collaboration.

## Closing Remarks

**Dr. McLemore:** As the Editor-in-Chief of *Health Equity*, I am so grateful for this rich discussion. Working closely with the guest editors for this special collection, we have put forward a rich array of articles that should assist clinicians, people with lived experience, students, researchers, and policymakers, and teachers should be able to use to activate new ideas and new ways of creating programs and uplifting community generated solutions. These pieces range from leveraging existing infrastructure (HEDIS measures) to developing site specific tools that can be adapted to other environments. I appreciate all of the effort and support that went into this special collection of papers and am grateful to the Commonwealth Fund for their generous support.

## Expert Panel

**Morenike Ayo-Vaughan, MS**, joined the Commonwealth Fund as program officer for the Advancing Health Equity team in 2021. She is responsible for supporting the vice president of Health System Equity, who leads the Advancing Health Equity program, and coordinates the overall work and efficiency of the team. Most recently, Ayo-Vaughan served as senior manager at Avalere Health, where she was responsible for serving myriad health care clients who sought the advancement of health equity and improvement in patient access to optimal care. She also was responsible for conducting literature reviews and key informant interviews and synthesizing findings and feedback from the advisory committee to inform the goals and strategies of Avalere s Health Equity Fund. Prior to Avalere, Ayo-Vaughan served as practice operations associate at Privia Health. She has an MS in health systems administration from Georgetown University and a BS in public health from Temple University.

**Dr. Andrew Bossick** received his PhD in Health Services Research from the University of Washington-Seattle in 2021. Dr. Bossick completed a 2-year postdoctoral fellowship at Henry Ford Health in the Department of Public Health Sciences focusing on women's health and racialized disparities. He joined the Department of Public Health Sciences at Henry Ford Health as an Assistant Scientist in 2023 and has been an Assistant Professor in the Departments of Obstetrics, Gynecology & Reproductive Biology at Michigan State University in the College of Human Medicine since 2022.

**Dr. Allison Bryant** is the Associate Chief Health Equity Officer for the Mass General Brigham integrated health system, developing and enacting MGB's United Against Racism campaign and its Health Equity Roadmap. She is an Associate Professor of Obstetrics, Gynecology and Reproductive Biology at Harvard Medical School. She received degrees in biology, public health and medicine from Harvard University, where she also completed training in Obstetrics and Gynecology and fellowships in Maternal Fetal Medicine and Minority Health Policy. She previously served as the Vice Chair for Quality, Equity and Safety in the Department of Obstetrics and Gynecology at Mass General Hospital. Dr. Bryant serves as a member of several regional and national women's health efforts; among them, the Massachusetts' Maternal Mortality Review Committee, which she chairs, ACOG's Clinical Consensus—OB Committee, for which she is Vice Chair, and the Program Committee for the Society for Maternal Fetal Medicine's Annual Pregnancy Meeting.

**Dr. Hector P. Rodriguez** is the Kaiser Permanente Endowed Professor of Health Policy and Management and Director of the Center for Healthcare Organizational and Innovation Research (CHOIR) at the UC Berkeley School of Public Health. He is an expert in organization behavior, performance management, and racial and ethnic disparities in health care. Dr. Rodriguez has authored and co-authored over 150 peer-reviewed publications. Prior to his academic career, he served as a management consultant for The Permanente Medical Group where he worked with leaders and clinicians to implement and evaluate organizational changes aimed at improving access and quality. Dr. Rodriguez is an elected member of the National Academy of Medicine and a recipient of the Thompson Prize from the Association of University Programs in Health Administration and the Distinguished Investigator Award from the AcademyHealth Disparities Interest Group.

**Dr. Eve Higginbotham**, inaugural Vice Dean for Inclusion, Diversity, and Equity of the Perelman School of Medicine at University of Pennsylvania is also a Senior Fellow at the Leonard Davis Institute for Health Economics and a tenured Professor of Ophthalmology at the University of Pennsylvania. She is an elected member of the NAM Council and member of the AAAS. Dr. Higginbotham is also a member of the Board of Directors of Ascension, chairing the Quality Committee for the system. She previously served terms on the Harvard Board of Overseers and the MIT Corporation. A graduate of MIT with SB and SM degrees in chemical engineering and MD from Harvard Medical School, Dr. Higginbotham completed her residency in ophthalmology at LSU and a glaucoma fellowship at the Massachusetts Eye and Ear Infirmary in Boston. In 2020, she completed a Master in Law degree from the University of Pennsylvania Carey Law School.

**Dr. Amanda Noroña-Zhou** is the Assistant Director of the Intergenerational Developmental Health Program at UCSF, a research scientist, and a licensed clinical psychologist. Her research interests include prenatal and intergenerational influences on child and family wellbeing and the role that community-level and systems-level factors can play in buffering families from stress, as well as addressing root causes of stress. In addition, Dr. Noroña-Zhou is committed to the translation of well-established psychological science findings into policies and programs that support the healthy development of children, families, and communities. Dr. Noroña-Zhou completed her PhD in Clinical Psychology from UCLA, her clinical residency in Child Health Psychology at Children's Hospital Colorado, and a NIMH-funded T32 postdoctoral fellowship at the University of Colorado Anschutz Medical Campus. In her spare time, Amanda enjoys baking chocolate chip cookies, reading POC-centered sci-fi/fantasy, and camping by any of California's beautiful lakes!

**Dr. Molly Richardson** is an assistant professor in the Department of Health Policy and Organization at the University of Alabama at Birmingham School of Public Health. Dr. Molly Richardson's work is focused on understanding and addressing health inequities. She was awarded the Charles Barkley Health Disparities Investigator Award during her formative studies for community-based participatory work in urban and rural communities in the Deep South. She enjoys working with multidisciplinary groups to conduct mixed methods research to identify ways in which inequities in access to care and quality services affect the reproductive health of minoritized people. Her recent qualitative work examined barriers to meaningful change and progress in achieving maternal health equity as well as emphasized contextually appropriate solutions. She applies the lessons from research to train future public health leadersg.

**Dr. Laurie Zephyrin** is the Senior Vice President of Advancing Health Equity at The Commonwealth Fund. Driven by a passion to transform health care, she brings her experience as a clinician, health policy maker, and intrapreneur to drive system transformation. She was the first national director of the Reproductive Health Program at the Department of Veterans Affairs, spearheading the strategic vision and leading systems changes to improve the health of women veterans nationwide and served as Acting Deputy Under Secretary for Health for Community Care. She was named a White House Fellow, Young Global Leader, Robert Wood Johnson Clinical Scholar and Aspen Health Innovator Fellow. Laurie earned her MD from the New York University School of Medicine, MBA and MPH from Johns Hopkins University, and BS in Biomedical Sciences from the City College of New York.
